# HIV-Associated Central Nervous System Disease in Patients Admitted at the Douala General Hospital between 2004 and 2009: A Retrospective Study

**DOI:** 10.1155/2013/709810

**Published:** 2013-02-26

**Authors:** Henry Namme Luma, Benjamin Clet Nguenkam Tchaleu, Elvis Temfack, Marie Solange Doualla, Daniela Pamela Ntchankam Ndenga, Yacouba Njankouo Mapoure, Alfred Kinyuy Njamnshi, Vincent-de-Paul Djientcheu

**Affiliations:** ^1^Department of Internal Medicine, Douala General Hospital, P.O. Box 4856, Douala, Cameroon; ^2^Faculty of Medicine and Biomedical Sciences, University of Yaoundé 1, Yaoundé, Cameroon; ^3^Université des Montagnes, Bangangte, Cameroon; ^4^Faculty of Medicines and Pharmaceutical Sciences, University of Douala, Douala, Cameroon; ^5^Yaoundé Central Hospital, Yaoundé, Cameroon

## Abstract

*Background*. Studies on HIV-associated central nervous system (CNS) diseases in Cameroon are rare. The aim of this study was to describe the clinical presentation, identify aetiological factors, and determine predictors of mortality in HIV patients with CNS disease. *Methods*. From January 1, 2004 and December 31, 2009, we did at the Douala General Hospital a clinical case note review of 672 admitted adult (age ≥ 18 years) HIV-1 patients, and 44.6% (300/672) of whom were diagnosed and treated for HIV-associated CNS disease. *Results*. The mean age of the study population was 38.1 ± 13.5 years, and median CD4 count was 49 cells/mm^3^ (interquartile range (QR): 17–90). The most common clinical presentations were headache (83%), focal signs (40.6%), and fever (37.7%). Toxoplasma encephalitis and cryptococcal meningitis were the leading aetiologies of HIV-associated CNS disease in 32.3% and 25% of patients, respectively. Overall mortality was 49%. Primary central nervous system lymphoma (PCNSL) and bacterial meningitis had the highest case fatality rates of 100% followed by tuberculous meningitis (79.8%). Low CD4 count was an independent predictor of fatality (AOR: 3.2, 95%CI: 2.0–5.2). *Conclusions*. HIV-associated CNS disease is common in Douala. CNS symptoms in HIV patients need urgent investigation because of their association with diseases of high case fatality.

## 1. Introduction

HIV infection is a major cause of morbidity and mortality worldwide and affects 33 million people of whom two-thirds live in sub-Saharan Africa [1]. The clinical presentation is diverse and many organ systems are involved. Its predilection for the nervous system makes it neuroinvasive, can enter the central nervous system (CNS), neurotropic, can live in neural tissues and neurovirulent, and can directly cause disease of the nervous system [2, 3]. This complex HIV-nervous system interaction therefore makes neurological manifestations a frequent complication of HIV. Nervous system disease is the main presenting feature in 10–20% of cases, and over 50% of patients with AIDS have neurological disease in the course of HIV disease [4]. It has also been shown that at autopsy 75–90% of HIV patients have neuropathologic abnormalities [4]. Neurological complications in HIV are highly disease stage specific, and this stage specificity largely reflects the dominant influence of altered immune responses especially cell-mediated defences that characterise later phases of systemic infections [5]. Since the introduction of highly active antiretroviral therapy (HAART) in 1996, the incidence of HIV-associated CNS disease has decreased in high income settings [6–11], even though mortality still remains high [12]. More so in resource limited settings where HAART scale-up is slow, most patients still present with severe immune-depression [13]. In addition, resource limited settings lack adequate diagnostic and therapeutic tools for HIV-associated CNS disease. In Cameroon where HIV prevalence among adults is estimated at 5.3% [1], and HAART coverage is low [14], the magnitude of HIV-associated CNS disease has been sparingly elucidated [15, 16]. We therefore decided to carry out a hospital-based retrospective clinical chart review of patients admitted with HIV-associated CNS disease in order to describe their clinical presentations and identify the different aetiologies, in hospital outcome and their associated factors.

## 2. Methods

### 2.1. Study Setting and Patients

We carried out this study at the Douala General Hospital, a tertiary referral centre in Douala, the largest city and economic capital of Cameroon. This hospital has a capacity of 320 beds and is the most specialised in the national subregion. Prior to commencing the study, hospital local institutional ethical clearance was sought and obtained. We included, all adult (age ≥ 18 years) HIV-1 positive patients were diagnosed with CNS disease (as described subsequently) between January 1, 2004 and December 31, 2009. Patient selection was done using the hospitalisation register of the Internal Medicine Unit of the Douala General Hospital, where the diagnosis and outcome of all hospitalised patients (discharged or dead) are recorded. Being a specialist hospital, the diagnosis of each patient is recorded by the specialist physician catering for patient. For the study subjects, the diagnoses were those of the neurologist. From this register patients diagnosed with HIV-associated CNS disease were sorted, and their files obtained from the archives for the collection of information relevant to the study. All patients with signs and symptoms evocative of HIV-associated CNS diseases but who had no clearly stated working diagnosis were excluded from the study. At the Douala General Hospital, HIV diagnosis is made according to the Cameroon National AIDS Control Programme guidelines [17] by antibody detection on two successive samples using a third-generation ELISA test BIOREX (Biorex Diagnostics Limited, Antrim, UK). In case of both samples are positive, a third sample is collected and tested using Genie III HIV-1/HIV-2 Assay (Bio-Rad Diagnostics, Marnes la Coquette, France) to specify either HIV 1 or HIV 2. The patient is declared to be positive for HIV if these three tests are positive. In case of any discordance, testing is done using western blot (New LAV blot, Diagnostics, Pasteur, Marnes la Coquette, France). 

### 2.2. Diagnosis of CNS Disease

 In this institution, the diagnosis of CNS diseases follows an algorithm. When a patient presents with symptoms of CNS disease with or without focal signs, a computerised tomographic (CT) brain scan is first done to exclude any space occupying lesions and/or signs of raised intracranial pressure (ICP). In the absence of these or any other finding that might contraindicate a lumbar puncture (LP), an LP is done for cerebrospinal fluid (CSF) analysis and culture. CSF analysis comprises doing a differential white blood cell count, measuring protein and glucose levels by standard biochemical methods, gram and Indian ink staining, and culture for pyogenic microorganisms. The working diagnosis is then made using an association of clinical, radiological, and/or biochemical findings as described subsequently.

The diagnosis of toxoplasma encephalitis (TE) was presumed by clinical presentation of fever, headache, and/or focal signs of sensory or motor deficits and an associated CT scan image of single or multiple intracerebral ring-enhancing lesions. In instances where CT scan could not be afforded, this diagnosis was retained when symptoms and clinical signs regress after commencing antitoxoplasma treatment which was usually cotrimoxazole.

Cryptococcal meningoencephalitis was diagnosed based on complaints of fever, headache, signs of meningeal irritation (stiff neck, Kernig or Brudzinski signs), and CSF finding of *Cryptococcus* on Indian ink stain.

Tuberculous meningitis diagnosis was presumed by the presence of fever, headache, signs of meningeal irritation with or without focal signs, and in CSF: elevated proteins, low glucose levels, negative pyogenic bacterial culture or antigen assay, past or present history of tuberculosis (TB), and/or suggestive chest X-ray findings or brain CT scan features suggestive of TB meningitis (basal meningeal enhancement, hydrocephalus, cerebral infarcts, oedema, and nodular enhancing lesions). However, this diagnosis was also considered when the patient did not improve on conventional antibiotics for pyogenic meningitis. 

AIDS dementia complex (ADC) was diagnosed by exclusion in patients with cognitive impairment after ruling out confounding conditions.

Diffused encephalitis include fever, altered consciousness, seizures, and diffuse cerebral oedema without focal signs.

Bacterial meningitis was diagnosed by detection of bacteria on gram stain or culture in CSF or positive test for bacterial antigens.

Primary central nervous system lymphoma (PCNSL) was diagnosed if the patient presented with headache, focal signs, altered behavior, intracerebral lesion with unifocal nodular heterogeneous contrast medium enhancement, and mass effect with surrounding oedema. This diagnosis was also made when a patient with suspected toxoplasmosis did not improve on treatment. 

### 2.3. Study Methods

We carried out a cross-sectional study on the files of patients admitted during the study period. Sociodemographic information, symptoms, and clinical signs relevant to CNS diseases, CD4 cell counts, and inpatient outcome (death or discharged) were obtained from these files and entered into a data base created using Epi Data version 3.1. Due to unavailability of information on antiretroviral treatment for most patients, it was not included on the data collection form.

### 2.4. Statistical Analysis

Data analysis was done using STATA 11.2 statistical package (Stata Corporation, College Station, TX, USA). The primary outcome of interest was in hospital death expressed as a percentage of the study population to obtain the overall mortality of HIV-associated CNS disease. Case fatality rates were then obtained as a percentage of deaths per diagnosis. Continuous variables were expressed using means and standard deviations where necessary, medians, and interquartile range (IQR). The Chi-square and Fisher's exact tests were used to compare categorical variables. Mantel Haenszel analysis was used to determine the strength of associations between the primary outcome and covariates, a logistic regression model was then built with covariates strongly associated with the outcome. Results of Mantel Haenszel analysis and logistic regression were reported as crude and adjusted odd ratios (OR), respectively, together with their 95% confidence interval (CI). Evidence of association was considered for a two-tailed *P* value of less than 0.05.

## 3. Results

During the study period, 20.4% (672/3299) of all admitted adult patients were HIV-1 infected, 300 of whom who fulfilled eligibility giving a prevalence of HIV-associated CNS disease were 44.6%. Among studied patients, and 54% (162) were males (Table 1). Mean age was 38.1 ± 13.5 years, and median CD4 cell count was 49 cells/mm^3^ (IQR: 17–90) with males having lower median CD4 counts than females (23 cells/mm^3^ (IQR: 11–46) versus 94 cells/mm^3^ (IQR: 53–180)), *P* < 0.001. Of all 300 patients, 77.7% (233) had a CT scan done, of whom 63.5% (148) subsequently had an LP. A total of 66.3% (199) of all patients had an LP done, but it was contraindicated in 33.7% (101) : 80.2% (81) of which was because CT scan showed space occupying lesion and 19.8% (20) due to other reasons. Macroscopically, 40.7% (81/199) of CSF were turbid. Median CSF proteins, glucose, and white cell count (WCC) were 1.2 g/L (IQR: 0.8–1.9), 0.4 g/L (IQR: 0.2–0.5), and 32 cells/mL (IQR: 12–55), respectively. Patients with Meningeal TB had the highest median protein levels, 1.7 (IQR: 1.3–2.2, *P* < 0.001) and Mycobacteria were found in the CSF of 1.9% (1/54) of patients with TB after Ziehl-Nielsen staining. *Listeria monocytogenes *was the most common germ found in 40% (3/5) of those with bacterial meningitis. Main CT scan findings included: cerebral oedema 55.8% (130/233), ring enhanced lesions 34.8% (81/233), diffused contrast enhancement 14.2% (33/233), cerebral atrophy 3.9% (9/233), and nodular enhancement 1.3% (3/233). The most common clinical presentation was headache in 83% (249/300) of patients, 30% (90/300) of which were associated with toxoplasma encephalitis (TE) (Table 2). Toxoplasma encephalitis and cryptococcal meningitis were the leading aetiologies of CNS disease (Table 3). The overall mortality in our study population was 49% (147) with bacterial meningitis and PCNSL which are responsible for the highest case fatality rates (Table 4); men being more likely to die than women (OR 14.9; 95% CI 7.4–30.2; *P* = 0.001). Low CD4 count, the presence of headache, focal signs, seizures, and signs of meningeal irritation are associated with death, but this association disappeared after adjusting for sex and low CD4 cell count (Table 5). Adjusting for sex, low CD4 cell counts remained strongly associated with death (Table 5). Patients with CNS disease who died had lower median CD4 count than those who survived (21 cells/mm^3^ (IQR: 10–43) versus 82 cells/mm^3^ (IQR: 51–167), *P* < 0.001). Though fever occurred in only 37.7% (113), 45.1% (51) of all those who died had fever (Pearson chi square = 27.7, *P* = 0.001). Coma present in 6.7% (20) was associated with a 100% case fatality rate (Tables 2 and 4).

## 4. Discussion

The prevalence of CNS disease observed during our study period was 44.6%, similar to that reported in Nigeria: 42.5% in 2005 [18] and Brazil: 46.5% in 2006 [13]. A lower prevalence of 21.6% [19] was reported in one study in Kenya, in 2007, a setting similar to ours. With available evidence that neurological complications in HIV are common and increase in frequency with severity of immune depression [4] this disparity in prevalence across similar settings might portray differences in HIV/AIDS disease burden and the challenges in the diagnosis of HIV-associated CNS disease. Though we found a high prevalence of CNS disease during our study period when HIV treatment scale-up was still at the primary stage, we speculate that present prevalence of HIV-associated CNS disease may still be high, because present national HAART scale-up barely approaches 60% [14], and present CNS disease diagnostic ability across the country has improved recently by the installation of CT scan machines in many regions. Nevertheless, we need more studies to depict this. 

Clinically, the most common presenting complaint in our study population was headache. Headache being a symptom present in overt brain dysfunction and meningitis [5] should always be properly investigated in HIV-infected patients most especially, as it could be the only symptom in life threatening CNS disease. In our study, it was the only complaint in 97.3% of patients with cryptococcal meningitis. Other studies also found headache to be common in 60–90% of patients with HIV-associated CNS disease [20, 21]. Meningeal signs which were present in 32% of patients were very common in bacterial, tuberculous, and cryptococcal meningitis. This is worth considering in resource limited settings where most diagnoses rely on patients' complaints and clinical findings. Therefore in areas where minimal CSF analysis is possible, clinicians should not hesitate to perform LPs in patients who present with these symptoms and signs. In HIV, CNS diseases have diverse nonclassical presentations, such that the presence of classical clinical signs might be a sign of severity as shown by the high mortality associated with meningeal signs in our study. CNS disease should therefore be suspected in the presence of any nervous system symptom in HIV-infected patients. Focal signs were also common and this could be explained by the etiologic factors of which space occupying lesions were the most prevalent. Our findings are consistent with others [20, 21].

Toxoplasma encephalitis was the most common aetiology of CNS disease in our study. Evidence shows that about a third of the world's population is chronically infected with *Toxoplasma gondii *[22]. Toxoplasma encephalitis in HIV which is a reactivation of quiescent chronic infection increases in frequency with severity of immune depression. In our study population 89% had a CD4 count of less than 200 cells/mm^3^ (similar to that in Ethiopia [21]), 57.7% of whom had less than 50 cells/mm^3^. Other studies had similar findings of severe immune depression in patients with HIV-associated CNS disease [13, 21]. Cryptococcal meningitis was the next leading cause of CNS disease in our study, similar to a Brazilian study [13], though they had a lower prevalence of 12.9%. In Ethiopia in 2012 [21] tuberculous meningitis was found to be the second leading cause of CNS disease, and though cryptococcal meningitis was the third, it had a prevalence similar to ours. This shows that even though HIV disease burden varies across settings, and the patterns of HIV-associated CNS diseases are similar.

AIDS dementia complex (ADC) was present in 2.7% of our patients. This finding is lower than what was found in similar African settings [18, 23]. Though evidence shows that neuropsychiatric impairment in HIV occurs in more than 50% of patients, most forms are mild or asymptomatic with severe disease occurring in only about 2% of patients [24]. Given the referral nature of our study setting, this could be a plausible reason why we found a lower prevalence because only patients with severe forms of ADC might have been referred to us. More so, with the existence of variable criteria of diagnosis and ascertainment [5], discrepancy in prevalence of ADC across settings should not be surprising. 

The overall mortality in our study population was 49% similar to that in Ethiopia [21] and Nigeria [18]. This shows that though CNS disease prevalence may vary across settings, the case fatality rate is similar. We also found men to be more likely to die than women, though HIV is more common in women than men in Cameroon [25]. This could be because men presented with much lower CD4 count which was an independent predictor of mortality. Existing evidence shows that there is a correlation between low CD4 count and the type of neurological disease [20], and the fact that we found men presenting with CNS diseases that had higher case fatality rates could also explain why more men were dying. In our study, though toxoplasma encephalitis was the most common aetiology of CNS disease, it was associated with the lowest case fatality rate. This could be because of the readily available and highly effective treatment. More so, in our setting, when an HIV patient presents with focal signs, most clinicians commence antitoxoplasma treatment, and a favourable response presumes the diagnosis especially in remote areas where CT scan is not readily available. Bacterial meningitis and PCNSL carried the highest case fatality rates. PCNSL fatality rates could be because of diagnostic difficulty and not readily available treatment. For bacterial meningitis, the high case fatality could be due to the fact that our institution is a reference hospital, and we receive severely ill patients who may have had some unsuccessful treatment that might have altered the clinical picture, thereby retarding our diagnosis and resulting in death. Tuberculous meningitis in our study had a fatality rate similar to that found in Nigeria [18] and in our milieu; one of the main explanations to this high fatality rate of TB meningitis is late diagnosis.

Though we showed that CNS disease prevalence is high among HIV patients in Douala, our study had certain limitations. The use of clinical case files for abstracting data for the study that rendered it difficultly for us to include other confounding causes of death in our analysis and missing data was a major flaw given that the clinical notes of all the patients are not reported the same way. More so, the exclusion of patients who might have had the disease conditions studied, but who did not have a working diagnosis, might have led to over or underestimation of causes of CNS disease in HIV. Furthermore, being a hospital-based study in an urban area, it may not capture the real picture of the burden of CNS disease in Cameroon. Finally, the absence of data on HAART during the study period when HAART availability was starting in sub-Saharan Africa might have heavily confounded mortality. However, our study has improved our knowledge on CNS disease in HIV patients in Cameroon and sets a template for further prospective studies in the field.

## 5. Conclusion 

HIV-associated CNS disease is common among HIV patients admitted to the Douala General Hospital. CNS symptoms in HIV patients require urgent investigation, as they might be associated with diseases that have high case fatality. In view of the high proportion of patients who still present in severe immune depression, more efforts are needed to reinforce guidelines pertaining to timely prophylaxis against opportunistic infections, early diagnosis, and treatment of CNS diseases, and commencement of HAART, as these would help reduce morbidity and mortality. 

## Figures and Tables

**Table 1:** General characteristics of 300 patients with CNS disease.

	*N* (%)
Age groups	
<30	93 (31.0)
30–39	87 (29.0)
40–49	50 (16.7)
50–59	51 (17.0)
>60	19 (6.3)
Sex	
Male	162 (54)
Female	138 (46)
Marital status	
Single	81 (27.0)
Married	197 (65.7)
Divorced	5 (1.7)
Widow(er)	17 (5.7)
CD4 groups	
<50	154 (51.3)
50–99	79 (26.3)
100–149	21 (7.0)
150–200	13 (4.3)
>200	33 (11.0)

**Table 2:** Frequency of clinical features of CNS disease in the study population (%).

	*N*	Head-ache	Focal Signs	fever	Meningeal signs	seizure	Altered sensorium	coma	Altered behaviour
Toxoplasma encephalitis	97	90	64	56	15	56	23	0	0
		(92.8)	(66)	(57.7)	(15.5)	(57.7)	(23.7)	(0.0)	(0.0)
Cryptococcal meningitis	75	73	26	0	47	0	0	0	0
		(97.3)	(34.7)	(0.0)	(62.7)	(0.0)	(0.0)	(0.0)	(0.0)
Diffused encephalitis	57	36	0	28	0	7	2	9	0
		(63.2)	(0.0)	(49.1)	(0.0)	(12.3)	(3.5)	(15.8)	(0.0)
Tuberculous meningitis	54	40	22	24	31	0	0	0	0
		(74.1)	(40.7)	(44.4)	(57.4)	(0.0)	(0.0)	(0.0)	(0.0)
ADC	8	6	6	0	0	0	4	6	8
		(75)	(75)	(0.0)	(0.0)	(0.0)	(50.0)	(75)	(100)
Bacterial meningitis	5	0	0	5	3	0	5	5	0
		(0.0)	(0.0)	(100)	(60.0)	(0.0)	(100)	(100)	(0.0)
PCNSL	4	4	4	0	0	0	0	0	4
		(1.6)	(3.3)	(0.0)	(0.0)	(0.0)	(0.0)	(0.0)	(100)

**Table 3:** Prevalence of the different aetiologies of CNS disease and the median CD4 cell count of 300 HIV positive patients.

Diagnosis	*N*	Prevalence	95% Confidence interval	Median CD4 (IQR)
Toxoplasma encephalitis	97	32.3	27.1–37.7	68 (43–103)
Cryptococcal meningitis	75	25.0	20.0–29.9	23 (10–61)
Diffused encephalitis	57	19.0	14.5–23.5	99 (49–204)
Tuberculous meningitis	54	18.0	13.6–22.4	16 (10–34)
AIDS dementia complex	8	2.7	0.8–4.5	15 (11–20)
Bacterial meningitis	5	1.7	0.2–3.1	8 (8–10)
PCNSL	4	1.3	0–2.6	16 (5–116)
Total	300	100	/	49 (17–90)

**Table 4:** In hospital mortality by aetiology in our study population of 300 HIV positive patients.

Diagnosis	*N*	Number of deaths (*n*)	Median CD4 count of fatal cases (IQR)	Case fatality rate % (*n*/*N*)
Toxoplasma encephalitis	97	29	41 (24–75)	29.9
Cryptococcal meningitis	75	39	12 (9–23)	52.0
Diffused encephalitis	57	22	45 (34–50)	38.6
Tuberculous meningitis	54	43	16 (10–31)	79.6
AIDS dementia complex	8	5	12 (9–14)	62.5
Bacterial meningitis	5	5	8 (8–10)	100
PCNSL	4	4	16 (5–116)	100
Total	300	147	21 (10–43)	49.0

**Table 5:** Univariate and multivariate (adjusting for sex and CD4 count) analysis of factors associated with death among 300 HIV positive patients with CNS diseases.

	OR	95% CI	*P* value	AOR	95% CI	*P* value
CD4 < 100	2.3	1.9–2.7	0.001	3.2*	2.0–5.2	0.001
Seizures	2.7	1.3–5.4	0.005	0.4	0.1–1.0	0.05
Headache	4.1	1.9–8.7	0.001	0.6	0.3–1.6	0.3
Focal signs	4.2	2.4–7.5	0.001	1.2	0.5–2.6	0.7
Meningeal signs	7.4	3.8–14.6	0.001	1.4	0.6–3.2	0.4

*Adjusted for sex.
